# Does the composition of urinary extracellular vesicles reflect the abundance of renal Na^+^/phosphate transporters?

**DOI:** 10.1007/s00424-022-02744-1

**Published:** 2022-09-08

**Authors:** Zsuzsi Radvanyi, Arezoo Daryadel, Eva Maria Pastor-Arroyo, Nati Hernando, Carsten Alexander Wagner

**Affiliations:** National Center of Competence in Research NCCR Kidney.CH, Institute of Physiology, University of Zurich, Winterthurerstrasse 190, CH-8057 Zurich, Switzerland

**Keywords:** Phosphate, Urinary extracellular vesicles, Slc34a1, Slc34a3

## Abstract

Studies addressing homeostasis of inorganic phosphate (Pi) are mostly restricted to murine models. Data provided by genetically modified mice suggest that renal Pi reabsorption is primarily mediated by the Na^+^/Pi cotransporter NaPi-IIa/*Slc34a1*, whereas the contribution of NaPi-IIc/*Slc34a3* in adult animals seems negligible. However, mutations in both cotransporters associate with hypophosphatemic syndromes in humans, suggesting major inter-species heterogeneity. Urinary extracellular vesicles (UEV) have been proposed as an alternative source to analyse the intrinsic expression of renal proteins in vivo. Here, we analyse in rats whether the protein abundance of renal Pi transporters in UEV correlates with their renal content. For that, we compared the abundance of NaPi-IIa and NaPi-IIc in paired samples from kidneys and UEV from rats fed acutely and chronically on diets with low or high Pi. In renal brush border membranes (BBM) NaPi-IIa was detected as two fragments corresponding to the full-length protein and to a proteolytic product, whereas NaPi-IIc migrated as a single full-length band. The expression of NaPi-IIa (both fragments) in BBM adapted to acute as well to chronic changes of dietary Pi, whereas adaptation of NaPi-IIc was only detected in response to chronic administration. Both transporters were detected in UEV as well. UEV reflected the renal adaptation of the NaPi-IIa proteolytic fragment (but not the full-length protein) upon chronic but not acute dietary changes, while also reproducing the chronic regulation of NaPi-IIc. Thus, the composition of UEV reflects only partially changes in the expression of NaPi-IIa and NaPi-IIc at the BBM triggered by dietary Pi.

**Supplementary Information:**

The online version contains supplementary material available at 10.1007/s00424-022-02744-1.

## Introduction

Aberrant low or high plasma phosphate (Pi) possess a health risk as illustrated by the development, among other symptoms, of rickets/osteomalacia and muscle weakness in hypophosphatemic states whereas ectopic calcifications, hyperparathyroidism, and cardiovascular disease associate with hyperphosphatemia (for review see [45]. The kidney is the main organ responsible for maintaining adequate and constant levels of plasma Pi. Thus, renal reabsorption of Pi decreases in response to Pi overload whereas it increases as a reaction to hypophosphatemia. Transport of Pi across renal epithelial cells is mostly mediated by two members of the SLC34 family of Na^+^/Pi cotransporters that are expressed at the brush border membrane (BBM) of proximal tubules, namely NaPi-IIa/SLC34A1 [9] and NaPi-IIc/SLC34A3 [37], for review see [23]. Their abundance is under hormonal and metabolic control, with the effects of parathyroid hormone (PTH) [1, 8, 10, 18, 19, 31, 32, 39], fibroblast growth factor-23 (FGF23) [15, 20, 42, 46], and dietary Pi [6, 11, 18, 21, 22, 38, 44] among the best studied. Ultimately, high dietary Pi as well as PTH and FGF23 (both hormones produced in states of hyperphosphatemia or upon ingestion of Pi-rich diets) reduce renal reabsorption of Pi by downregulating the expression of cotransporters, whereas low dietary Pi triggers the opposite effects.

Gene ablation studies in mice suggested that NaPi-IIa has a major quantitative role [2], whereas the contribution of NaPi-IIc to renal Pi reabsorption in adult mice seems negligible [26, 40]. Furthermore, several reports in rodents also indicate that the expression NaPi-IIa adapts quickly to changes on Pi-regulating factors (including PTH and dietary Pi), whereas adaptation of NaPi-IIc is far slower [32, 44]. Based on these observations, NaPi-IIa was long considered as the main renal transporter. However, this view may not apply to humans since mutations in NaPi-IIc seem as severe as those in NaPi-IIa. Thus, while mutations of NaPi-IIa associate with idiopathic infantile hypercalcemia (IIH/HCINF2; OMIM: 616,963) [33, 36], mutations affecting NaPi-IIc cause hereditary hypophosphatemic rickets with hypercalciuria (HHRH; OMIM: 241,530) [3, 17, 24]. In both cases, hypophosphatemia caused by urinary Pi loss triggers a compensatory upregulation of 1,25(OH)_2_ vitamin D_3_ aimed at stimulating intestinal Pi absorption, that also results in higher absorption of Ca^2+^, hypercalcemia and hypercalciuria. Understandably, information about the role, regulation and contribution of single transporters to Pi homeostasis in humans is sparse and almost limited to reports describing the discovery of disease-causing mutations.

Urinary extracellular vesicles (UEV) have been proposed as a potential surrogate to analyse the expression of renal proteins in vivo, since several reports suggest that the content of transporters in UEV changes in parallel with their renal expression [14, 27–30, 43, 48, 49] (for review see [12, 35]). However, there are also studies suggesting a lack of correlation of transporter abundances in paired collections of human nephrectomy samples and UEV [34]. Here, were tested the reliability of UEV cargo as an indirect source to study regulation of renal Na^+^/Pi cotransporters in vivo. As a proof of principle, we have compared the abundance of NaPi-IIa and NaPi-IIc in paired samples of kidneys and UEV from rats acutely and chronically fed high or low Pi-diets.

## Material and methods

### Animal handling and sample collection

After 2 weeks of adaptation, male Wistar rats 10 weeks old with a body weight of approximately 200 g (Janvier Labs, France) were split into 2 groups of 10 animals each and fed 4 days on diets (Granovit AG, Switzerland) containing either high (H: 1.2% w/w) or low Pi (L: 0.1% w/w). Both diets had similar Ca^2+^ (1%) and vitamin D_3_ (800 μ/kg) content. On the fifth day, rats were single placed in metabolic cages (Tecniplast, Italy), and in the evening 5 animals from each group were switched from their initial diet to the opposite diet while the remaining 5 rats stayed on their starting chow. Urine was collected for the next 12 h (8 p.m.–8 a.m.) under mineral oil in the presence of EDTA-free protease inhibitors (Roche, Switzerland). Thus, this study consists of 4 experimental groups each containing 5 animals: rats chronically (5 days) fed on either high Pi (HH) or low Pi (LL) and rats acutely switched (12 h) to either high Pi (LH) or low Pi (HL). Urine samples were centrifuged at 163 × g for 10 min at room temperature and supernatants were stored at -20 °C (ion measurements) or at − 80 °C (isolation of UEV). After urinary collection, rats were anaesthetized with isoflurane and blood was withdrawn from the vena cava with heparinized syringes. Upon centrifugation of blood at 9300 × g for 8 min at 4 °C, plasma was aliquoted and stored at − 80 °C. Rats were sacrificed by cervical dislocation and their kidneys collected and stored at − 80 °C. Animal handling was previously approved by the local veterinary authority (Kantonales Veterinäramt Zürich), according to the Swiss Animal Welfare laws (licence number 156/2016).

### Concentration of Pi, Ca^2+^ and creatinine in plasma and urine

The concentration of Pi was measured with a kit based on the Fiske-Subbarow method (Randox, UK). The concentration of total Ca^2+^ was quantified with a Quantichrom Calcium Assay kit (Bioassay Systems, USA). The concentration of creatinine in plasma and urine was quantified with an enzymatic-based technique (SYNCHRON Systems) or with the Jaffe method (Wako Chemicals), respectively.

### Quantification of PTH, FGF23 and 1,25(OH)_2_D_3_ in plasma

Intact PTH and intact FGF23 were both measured using ELISA-based kits (Immutopics, USA). The plasma concentration of 1,25(OH)_2_D_3_ was quantified by radioimmunoassay (Immunodiagnostic System, UK).

### Isolation of renal BBM

Renal BBM were prepared according to the Mg^2+^-precipitation technique [5]. In brief, half of the kidneys were homogenized in ice-cold buffer using the Brinkmann Polytron PT 10/35 (Kriens, Switzerland). The homogenization buffer (300 mM D-mannitol, 5 mM EGTA, 12 mM Tris–HCl, pH 7.1) contained EDTA-free protease inhibitors. Upon addition of MgCl_2_ (final concentration 12 mM) and incubation for 15 min on ice, samples were centrifuged at 2600 × g for 15 min at 4 °C. Supernatants were centrifuged at 39,400 × g for 30 min at 4 °C, and pellets resuspended in experimental buffer (300 mM D-mannitol, 20 mM HEPES-Tris, pH 7.4). Samples were centrifuged again at 39,400 × g for 30 min at 4 °C, and the BBM-containing pellets were resuspended in the experimental buffer and stored at − 20 °C. Protein concentrations were measured with a commercial kit (Bio-Rad, Hercules, CA, USA).

### Isolation of UEV

The isolation of UEV was performed using standard procedures as previously described [16]. Briefly, 2 ml urine was centrifuged at 17,000 × g for 15 min at RT. Supernatants were kept at RT, while pellets were resuspended in isolation solution (250 mM sucrose, 10 mM triethanolamine, pH 7.6) containing 1.2 M dithiothreitol (DTT). After incubation for 5 min at 37 °C, pellets were vortexed until dissolved, and centrifuged at 16,000 × g for 15 min at 4 °C. These supernatants were pooled with those obtained after the first centrifugation and pools were subjected to ultracentrifugation at 200,000 × g for 2 h at 4 °C. Pellets containing the UEV were resuspended in 80 µl of 1.5 × Laemmli sample buffer containing 150 mM DTT and stored at − 20 °C.

### Western blot

BBM (20 µg) and UEV (amount equivalent to 7.5 µg urinary creatinine) were separated on 9–10% SDS-PAGE and proteins transferred to polyvinylidene difluoride membranes (Immobilon-P, Millipore, Switzerland). Blocking and incubation with primary/secondary antibodies was done in 5% fat-free milk powder/TBS (150 mM NaCl, 25 mM Tris base, pH 7.4) following standard procedures [9]. After exposure to substrate (Immobilon Western Chemiluminescent HRP Substrate, Millipore, Switzerland), signals were detected with the LAS-4000 Luminescent Image Analyzer (Fujifilm, Japan) and quantified with ImageJ. When required, membranes were stripped 15 min at RT in 25 mM glycine, 1% SDS, pH 2. Staining of total proteins (LiCor) was used for loading normalization of BBM (supplementary Figs. 1 and 2). Source and dilutions of primary and secondary antibodies are provided in Table 1.

**Table 1:** Source, features and dilutions of used antibodies

Antibody	Source	Features	Dilution
NaPi-IIa	Home made (Custer M et al., Am J Physiol. 1994)	Rabbit polyclonal Antigen: rat N-terminal 13 aa	1: 3000
NaPi-IIc	Home made (Nowik M et al., Pflügers Arch. 2008)	Rabbit polyclonal Antigen: rat C-terminal 13 aa	1: 1500
AQP2	Santa Cruz sc-515770	Mouse monoclonal Antigen: human C-terminal aa 232–271	1:2000
TSG101	Santa Cruz sc-7964	Mouse monoclonal Antigen: full-length mouse TSG101	1:500
HRP-a-rabbit	Promega #W4011	Goat polyclonal	1:5000
HRP-a-mouse	Promega #W4021	Goat polyclonal	1:5000

### RNA extraction and real-time PCR

Pieces of kidneys were homogenized in RTL buffer and RNA was purified (RNeasy Mini Kit; Qiagen Science, Germany) and reverse transcribed (TaqMan Reverse Transcription kit; ThermoScientific, USA) following the manufacturers’ instructions. The expression of the genes of interest was analysed by semi-quantitative real-time PCR using the KAPA Probe Fast qPCR Universal Master Mix (2x) Kit (Kapa Biosystems, South Africa) in the presence of 0.1 µM FAM/TAMRA-labelled probe and 1 µM primers (Table 2). qPCR was run in the 7500 Fast Real-Time PCR System (Applied Biosystems, Switzerland). Cycle thresholds (Ct) were manually set at the exponential phase of the amplification curve. Ct values of tested genes were normalized to hypoxanthine–guanine phosphoribosyltransferase (HPRT), with relative mRNA expression levels calculated as 2^(Ct_HPRT – Ct_gene).^

**Table 2:** Sequences of primers (forward/reverse) and probes used for qPCR

Gene	Primers/Probe
NaPi-IIa	Forward: GGA ATC ACA GTC TCA TTC GGA TT
	Reverse: ATG GCC TCT ACC CTG GAC ATA G
	Probe: TGT CAA CCA GAG ACA AAA GAG GCT TCC ACT
NaPi-IIc	Forward: GGG ATC GGG ATG AAT TTC AGA
	Reverse: GGG CCA GCT CAC TCA GTC TCT
	Probe: ACG GCA TCT TCA ACT GGC TCA CAG TGT T
HPRT	Forward: GCT GAA GAT TTG GAA AAG GTG TTT A
	Reverse: ACA CAG AGG GCC ACA ATG TGA
	Probe: TTA TGG ACA GGA CTG AAA GAC TTG CTC GAG ATG

Data are presented as scatter plots together with means ± SEM. Statistical significances were calculated by *t*-test or one-way ANOVA with Bonferroni’s multiple comparison test, as indicated, using Graph Prism version 8 (GraphPad Software, San Diego, CA, USA). *P* < 0.05 was considered significant.

## Results

### Urinary and plasma concentrations of Pi directly correlate with dietary Pi content

**Fig. 1:**
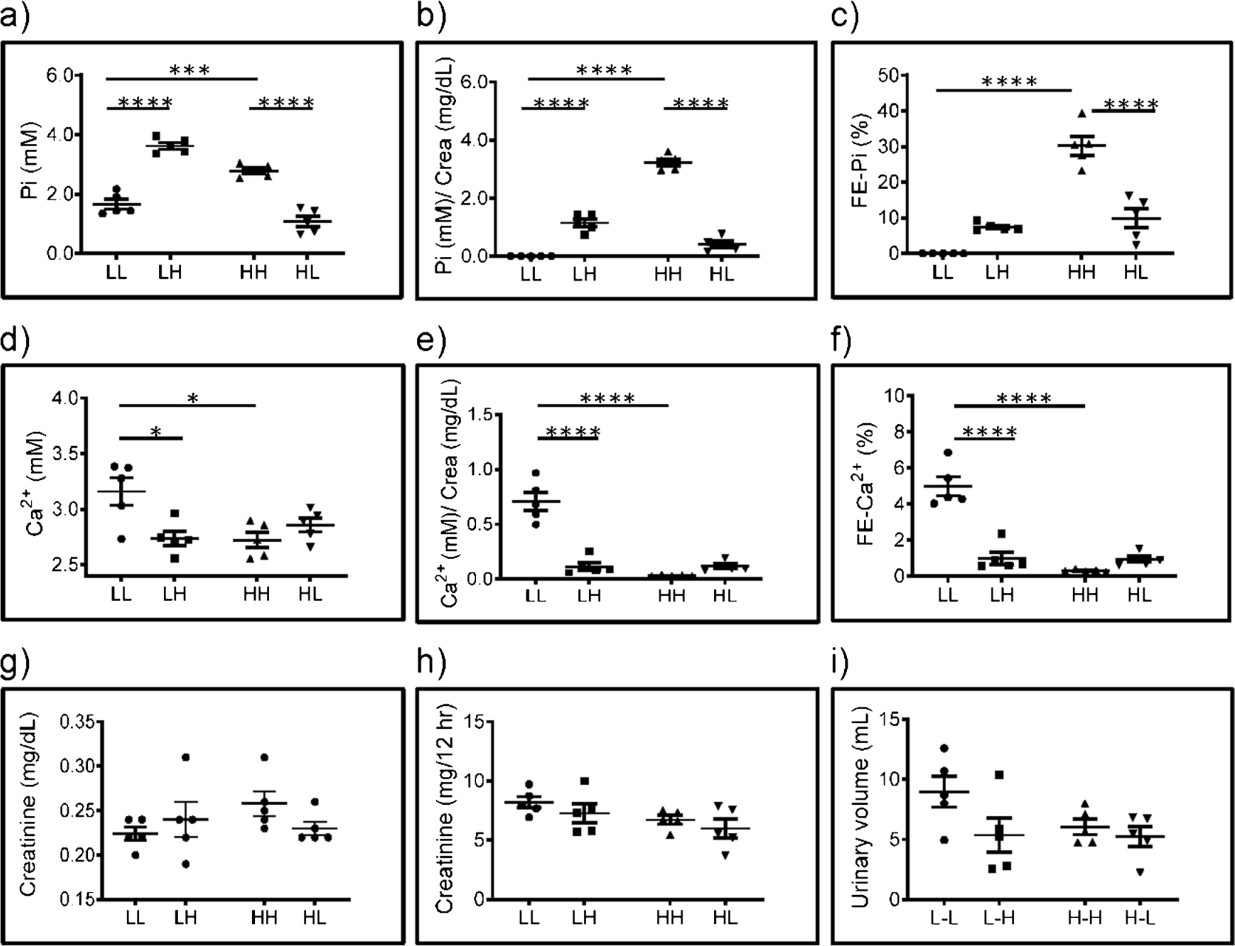
Urinary and plasma levels of Pi and Ca^2+^ correlate with dietary Pi content. (**a**) Plasma concentration of Pi, (**b**) urinary excretion of Pi, (**c**) fractional excretion of Pi, (**d**) plasma concentration of Ca^2+^, (**e**) urinary excretion of Ca^2+^, (**f**) fractional excretion of Ca^2+^, (**g**) plasma creatinine, (**h**) urinary excretion of creatinine and (**i**) urinary volume in samples from rats fed chronically (5 days) with low Pi (LL), acutely changed (12 h) from low to high Pi (LH), chronically fed with high Pi (HH) and acutely changed from high to low Pi (HL). Statistical significances were calculated with one-way ANOVA with Bonferroni’s multiple comparison test. n = 5 for each group, * *P* < 0.05, **P < 0.01, *** *P* < 0.001, **** *P* < 0.0001

As expected, the concentration of Pi in plasma was higher in rats chronically fed on high Pi (HH) compared with the low Pi (LL) group and correlated with dietary Pi also upon acute switches (Fig. 1a). Similarly, the urinary excretion of Pi (measured as Pi/creatinine ratio) was higher in rats chronically fed high Pi than in the group chronically fed low Pi (Fig. 1b). Furthermore, acute switches resulted in the expected higher (LL to LH) or lower (HH to HL) urinary Pi output. Plasma and urinary levels of creatinine (Fig. 1g, h) as well as urinary volume (Fig. 1i) were similar in all groups. The fractional excretion of Pi followed the same trend as the urinary Pi excretion, though the increase induced by the acute switch from low to high Pi did not reach statistical significance (Fig. 1c).

### Urinary and plasma concentrations of Ca^2+^ inversely correlate with dietary Pi content

Plasma concentration of Ca^2+^ was lower in rats acutely and chronically fed high Pi than in the corresponding low Pi groups (Fig. 1d). Consequently, the urinary excretion of Ca^2+^ was lower in rats chronically fed high Pi (HH) than in the group chronically fed low Pi (LL), and this difference was observed in both Ca^2+^/creatinine ratio (Fig. 1e) as well as fractional excretion (Fig. 1f). The acute change from low to high Pi (LL to LH) also resulted in lower urinary Ca^2+^, whereas the acute switch from high to low Pi (HH to HL) had no effect (Fig. 1e, f).

### Plasma levels of Pi-regulating hormones differentially adapt to changes on dietary Pi

**Fig. 2:**
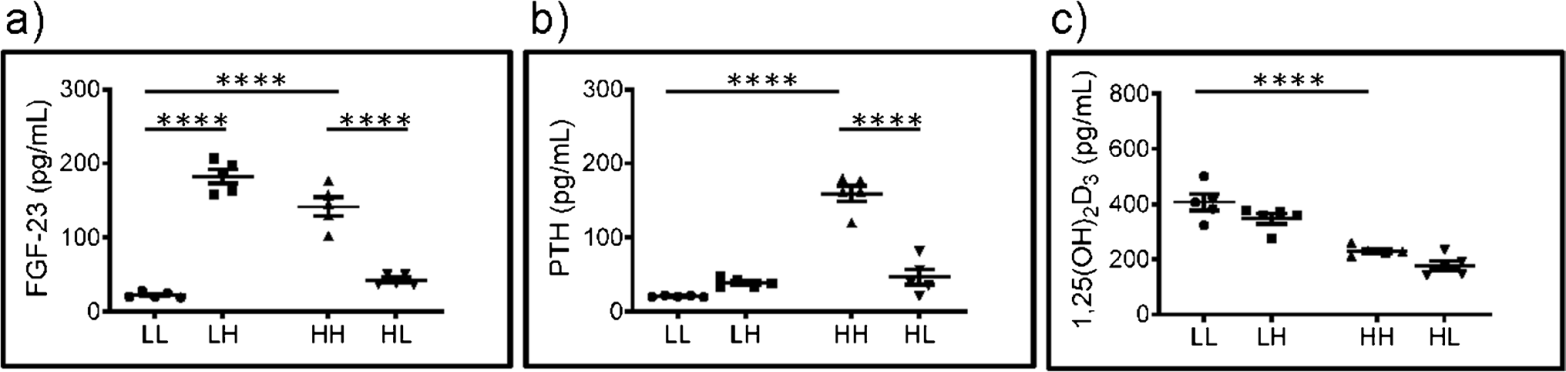
Plasma levels of Pi-regulating hormones differentially adapt to changes in dietary Pi. Plasma levels of (**a**) intact FGF-23, (**b**) intact PTH and (**c**) 1,25(OH)_2_ vitamin D_3_ in samples from rats fed chronically (5 days) with low Pi (LL), acutely changed (12 h) from low to high Pi (LH), chronically fed with high Pi (HH) and acutely changed from high to low Pi (HL). Statistical significances were calculated with one-way ANOVA with Bonferroni’s multiple comparison test. n = 5 for each group, **** *P* < 0.0001

Plasma levels of intact FGF-23 (Fig. 2a) and PTH (Fig. 2b) were higher in rats fed chronically high Pi (HH) than in those fed chronically low Pi (LL). Both phosphaturic hormones were also regulated in response to acute switches, increasing upon changing from low to high Pi (LL to LH) and decreasing upon switching from high to low (HH to HL) Pi, though the change in PTH induced by the first switch did not reach statistical significance. 1,25(OH)_2_D_3_ was lower in animals fed chronically high Pi (HH) compared with the group chronically fed on low Pi (LL) but did not change in response to acute changes (Fig. 2c).

### The abundance of the proteolytic fragment of NaPi-IIa in UEV partially correlates with its renal expression

**Fig. 3:**
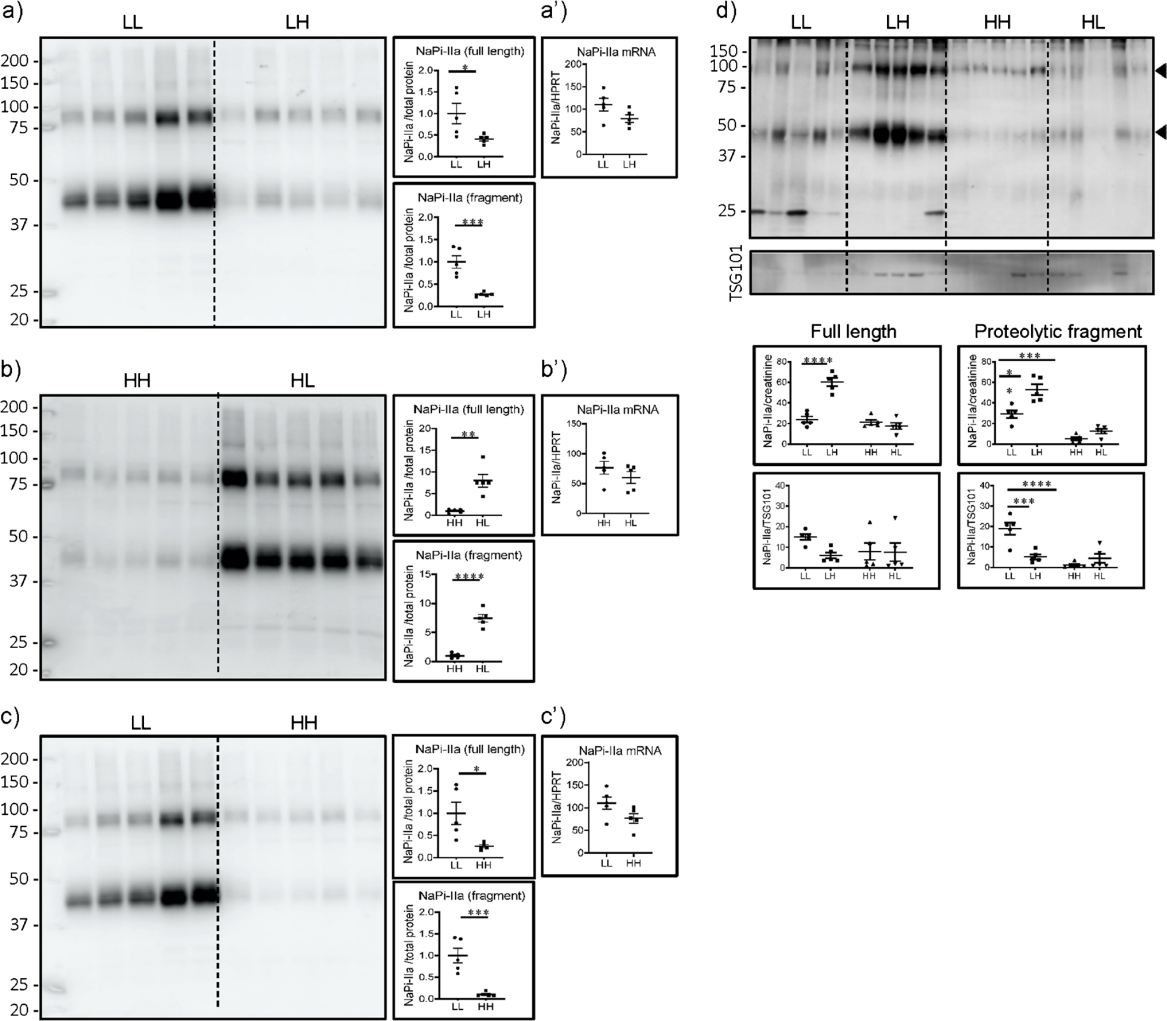
The abundance of the proteolytic fragment of NaPi-IIa in UEV partially correlates with its renal expression. Western blots for NaPi-IIa in renal BBM from rats fed (**a**) chronically (5 days) low Pi (LL) and acutely changed (12 h) from low to high Pi (LH), (**b**) chronically fed high Pi (HH) and acutely changed from high to low Pi (HL), (**c**) chronically fed low (LL) or high Pi (HH), and (**d**) UEV isolated from all 4 groups. Graphs show the quantifications of the full-length and proteolytic fragment normalized to (a–c) LiCor total protein stain (suppl Fig. 1), (**d**) urinary creatinine and TSG101. (**a’–c’**) real-time PCRs on renal RNA samples from the same groups. Statistical significances were calculated by *t*-test (a–c and a’–c’) or with one-way ANOVA with Bonferroni’s multiple comparison test (**d**). n = 5 for each group, *P < 0.05, **P < 0.01, *** *P* < 0.001, **** *P* < 0.0001. Red asterisks indicate changes in UEV similar to those described in renal BBM

As reported [4], incubation of renal BBM with the NaPi-IIa antibody resulted in the detection of a smear with an apparent molecular weight between 75 and 100 kDa, corresponding to the full length glycosylated protein, together with a lower smear between 37 and 50 kDa known to be a N-terminal proteolytic fragment which is also glycosylated (Fig. 3a–c). As expected [22, 44], the abundance of both bands was lower in BBM from rats acutely switched from low to high Pi (LH) than in rats maintained chronically on low Pi (LL), whereas their expression was higher in rats acutely switched from high to low Pi (HL) than in rats chronically kept on high Pi (HH) (Fig. 3a, b). Also as expected [22, 44], the expression of both fragments was lower in BBM from rats chronically fed on high Pi (HH) than in the chronic low Pi group (LL) (Fig. 3c). In all three cases, no differences on mRNA levels were detected between groups (Fig. 3a’–c’). Together with the mineral and hormonal data described above, these results indicate that the feeding protocols had triggered the expected systemic and renal adaptation.

Bands corresponding to the full-length and the proteolytic NaPi-IIa fragment were also detected in UEV (Fig. 3d). However, their quantification heavily relied on the methods used for normalization. Thus, the abundance of both fragments seemed surprisingly higher in UEV isolated from rats acutely switched from low to high Pi (LH) than in samples from rats chronically kept in low Pi (LL) upon normalization to creatinine, whereas no difference or even the opposite change was detected upon normalization to TSG101. However, TSG101 levels fluctuated within and between groups. Regardless of the method for normalization, the acute switch from high to low Pi (HH to HL) did not alter the content of either fragment in UEV. However, both normalization methods indicate that the abundance of the proteolytic product is lower in UEV from rats chronically fed high Pi (HH) than in the chronic low Pi group (LL), a finding in agreement with its expression in renal BBM (Fig. 3c). In contrast, the content of the full-length transporter was similar in UEV from both chronic groups.

### The abundance of NaPi-IIc in UEV partially correlates with its renal expression

**Fig. 4:**
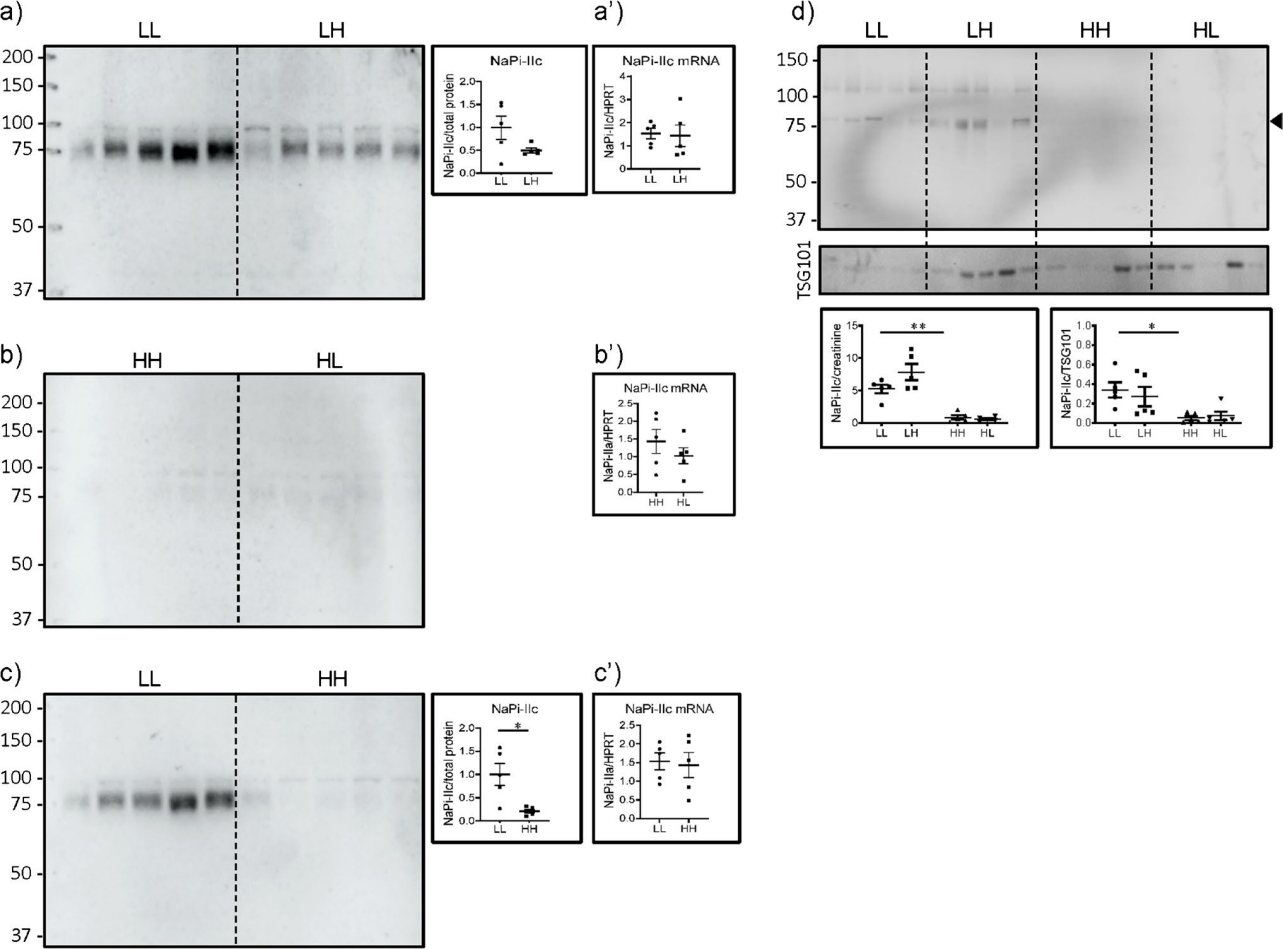
The abundance of NaPi-IIc in UEV partially correlates with its renal expression. Western blots for NaPi-IIc in renal BBM from rats fed (**a**) chronically (5 days) low Pi (LL) and acutely changed (12 h) from low to high Pi (LH), (**b**) chronically fed high Pi (HH) and acutely changed from high to low Pi (HL), (**c**) chronically fed low (LL) or high Pi (HH), and (**d**) UEV isolated from all groups. Graphs show the quantifications normalized to (a–c) LiCor total protein stain (supplementary Fig. 1), (**d**) urinary creatinine and TSG101. (a’–c’) real time PCRs on renal RNA samples from the same groups. Statistical significances were calculated by *t*-test (a–c and a’–c’) or with one-way ANOVA with Bonferroni’s multiple comparison test (d). n = 5 for each group, *P < 0.05, ** *P* < 0.01. Asterisks indicate changes in UEV similar to those described in renal BBM

The expression of NaPi-IIc, detected only as the full-length protein, tended to be lower in renal BBM from rats acutely switched to high Pi (LH) compared with rats chronically fed on low Pi (LL), though the difference did not reach statistical significance (Fig. 4a). Its expression was very low in kidneys from rats chronically fed on high Pi (HH), and acute switched to low Pi (HL) failed to increase substantially its expression (Fig. 4b). As expected, renal expression of NaPi-IIc was lower in rats chronically fed on high Pi (HH) compared with those kept chronically on low Pi (LL) (Fig. 4c). The chronic change did not involve transcriptional regulation since no differences in the mRNA expression of the cotransporter were found between groups (Fig. 4a’–c’).

NaPi-IIc was also detected in UEV, though its abundance was at the limit of detection with the available antibody (Fig. 4d). As in renal BBM, the amount of NaPi-IIc in UEV was lower in rats chronically fed on high Pi (HH) compared with animals kept chronically on low Pi (LL) using both normalization criteria.

### The abundance of AQP2 in UEV partially correlates with its renal expression

**Fig. 5:**
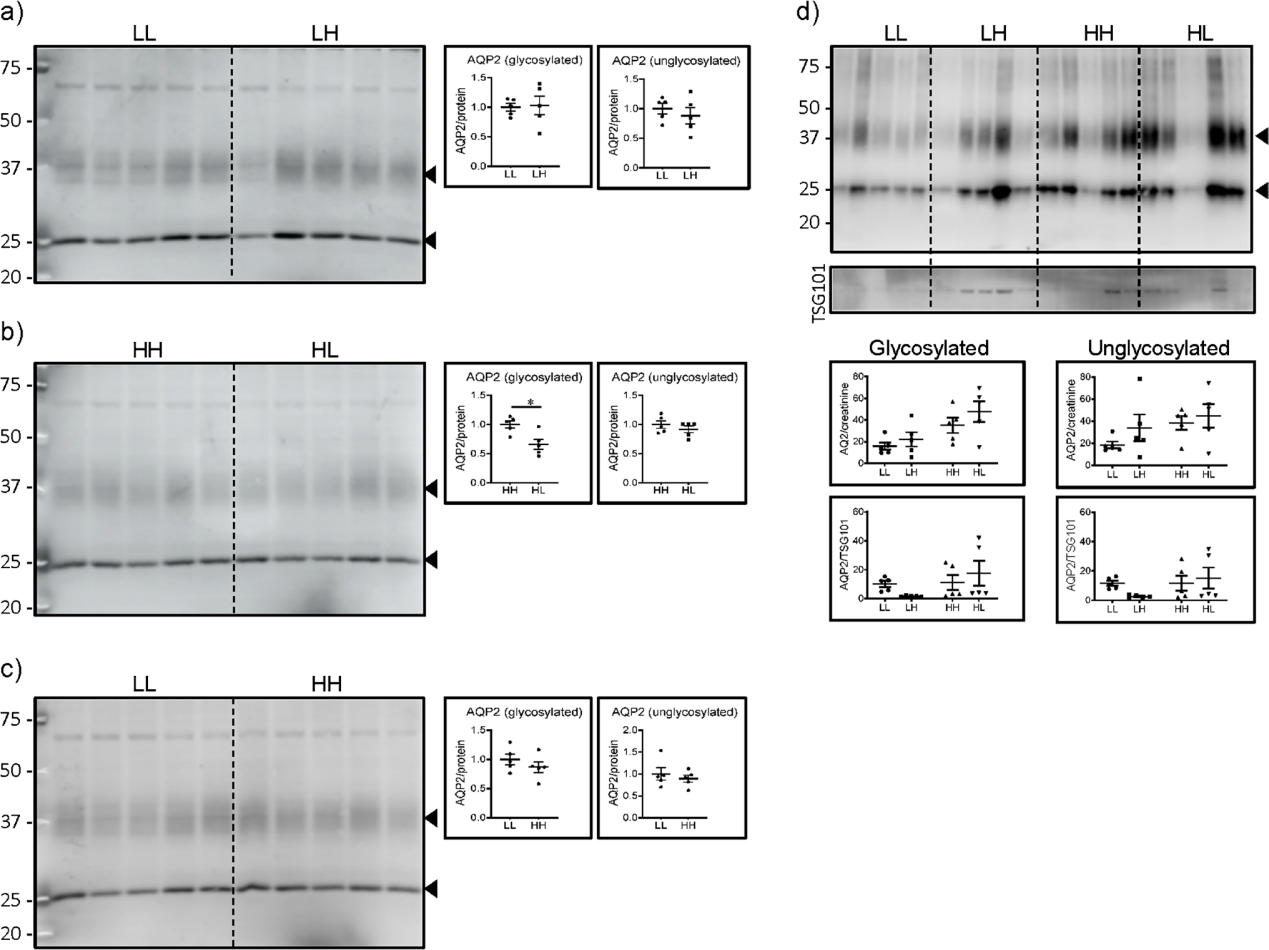
The abundance of AQP2 in UEV correlates with its renal expression. Western blots for AQP2 in renal BBM from rats fed (**a**) chronically (5 days) low Pi (LL) and acutely changed (12 h) from low to high Pi (LH), (**b**) chronically fed high Pi (HH) and acutely changed from high to low Pi (HL), (**c**) chronically fed low (LL) or high Pi (HH), and (**d**) UEV isolated from all 4 groups. Graphs show the quantifications of the unglycosylated and glycosylated channel normalized to (a–c) LiCor total protein stain (supplementary Fig. 2), (**d**) urinary creatinine and TSG101. Statistical significances were calculated by *t*-test (A–C) or with one-way ANOVA with Bonferroni’s multiple comparison test (D). n = 5 for each group, *P < 0.05

As a negative control for the dietary effect, we analysed the expression of the collecting duct water channel AQP2. This channel is detected as both glycosylated (smear at 37 kDa) and unglycosylated (25 kDa band) proteins [13]. As expected, its renal expression did not change in response to dietary Pi (Fig. 5a–c), except for a small reduction of the glycosylated form in rats acutely changed from high to low phosphate (Fig. 5b). Both forms were also detected in UEV, and their pattern of expression was similar in all dietary conditions (Fig. 5d).

## Discussion

The contribution of the Na^+^/Pi cotransporters NaPi-IIa/*Slc34a1* and NaPi-IIc/*Slc34a3* to renal reabsorption of Pi may have some species-specificity, since the consequences of ablation/mutation in mice and humans do not fully overlap [2, 3, 17, 24, 26, 33, 36, 40]. Still, all that is known about the regulation of the transporters in vivo is based on studies in rodent models. UEV, heterogenous vesicular structures that originated from different parts within the urogenital tract, have been proposed as an alternative source to study the expression and regulation of renal proteins (for review see [35]) and a recently published large scale proteomic analysis suggests that UEV cargo reliably reflects changes in renal protein expression [49]. Based on comparative analysis of Western blots of purified UEV and renal samples, the content of several transporters in UEV has indeed been shown to change in parallel to their expression in renal tissue in response to physiological regulators. This includes the abundance and regulation of total and phosphorylated NCC (the thiazide-sensitive NaCl cotransporter located in distal convoluted tubules) [28, 43, 48], $\gamma$ENaC (the amiloride-sensitive epithelial Na^+^ channel expressed in collecting ducts) [14], pendrin (an anion exchanger in cortical collecting ducts) [27, 30], and the B1 subunit of the V-ATPase (a H^+^-ATPase expressed in intercalated cell) [29]. However, discrepancies between the protein content of UEV and renal samples have also been described. Thus, no correlation of the levels of NaPi-IIa, the outer medullary potassium channel ROMK, NCC, and the water channel aquaporin 2 (AQP2) was found in paired collections of human nephrectomy samples and UEVs [34].

Here, we explored the possibility of using UEV to study regulation of renal Na^+^/Pi cotransporters in vivo following standard procedures used by others to study in vivo regulation of renal proteins [27–30, 34, 43, 48]. We choose the same technical approach (purification of UEV followed by Western blot) to better compare our results with published data. For that, and as a proof of principle, we have compared the abundance of NaPi-IIa and NaPi-IIc in paired samples of kidneys and UEV from rats subjected to a procedure known to induce robust changes in the renal expression of both cotransporters, namely feeding with diets with either high or low Pi [6, 11, 18, 21, 22, 38, 44]. In order to test how dynamic changes in renal expression could be reflected in UEV content, diets were provided chronic and acutely. We document that the dietary interventions resulted in the expected systemic changes. Thus, hyperphosphaturia and hyperphosphatemia were observed in the groups acutely and chronically fed with high Pi compared with those fed with low Pi, whereas hypercalciuria and hypercalcemia developed in rats chronically fed on low Pi, the last finding at least partially explained by enhanced intestinal Ca^2+^ absorption due to less formation of Ca^2+^-phosphate precipitates and higher circulating levels of 1,25(OH)_2_ vitamin D_3_. Plasma levels of FGF-23 and PTH were higher in the groups fed with high Pi than in those fed with low Pi, as expected based on the hyperphosphatemia associated with dietary Pi overload. In agreement with the hormonal data, renal expression of NaPi-IIa and NaPi-IIc was lower in rats chronically fed with high Pi, whereas acute changes resulted in regulation of only NaPi-IIa, consistent with the known slower response of NaPi-IIc to dietary (and PTH) changes [32, 44].

NaPi-IIa and NaPi-IIc were both detected in UEV. However, quantification and comparison of their abundance among the four experimental groups were strongly dependent upon the normalization method. Urinary creatinine has been suggested as one of the most reliable normalization criteria, since it may correct for effects of circadian rhythm and for changes in glomerular filtration rate [12]. Because comparable levels of urinary (and plasma) creatinine as well as urinary output volumes were found in all analysed groups, gels were loaded with quantities of UEV corresponding to equal amounts of urinary creatinine. Normalization to creatinine resulted in a paradoxical higher abundance of the full-length and proteolytic fragment of NaPi-IIa (and a similar tendency of NaPi-IIc) in UEV isolated from rats acutely switched to high Pi (LH) compared with the group chronically fed low Pi (LL). However, it properly reflected the renal reduction of the proteolytic NaPi-IIa fragment as well as of the abundance of NaPi-IIc induced by chronic dietary Pi load (HH vs LL). The product of the tumor susceptibility gene 101 (TSG101), a regulatory component of vesicular trafficking required for sorting of endosomes into multivesicular bodies (MVBs) [25], is one of the proteins often used to normalize UEV samples. Normalization to TSG101, whose abundance was highly heterogeneous in rat UEV, eliminated the apparent increase of full-length NaPi-IIa and even resulted in a reduction of the proteolytic fragment in UEV from acutely Pi-loaded rats. Instead, it still reflected the lower renal expression of the NaPi-IIa proteolytic fragment as well as of NaPi-IIc in rats chronically fed on high Pi as compared with the chronic low Pi group. In this regard, it may be worth to indicate that EVs contain two types of secreted vesicles, namely exosomes (generated by fusion of MVBs with the plasma membrane) and microvesicles (generated by direct budding of the plasma membrane), as well as apoptotic bodies (for review see [12, 41]). Furthermore, the abundance of TSG101 (as well as that of other routinely used UEV biomarkers such as programmed cell death-6 interacting protein PDCD6IP/ALIX and heat shock protein 70/HSP70) seems to be higher in exosomes than other UEV components [7]. On the other hand, it is known that in response to high dietary Pi, NaPi-IIa is internalized into clathrin-coated pits and via endosomes is targeted to lysosomes for degradation [11]. Although speculative, overrepresentation of exosomes in the UEV preparations could explain the paradoxical higher abundance of NaPi-IIa in UEV isolated from rats acutely switched to high Pi observed upon normalization to creatine as well as the elimination of this difference upon normalization to TSG101. Another speculation may be, that removal of cleaved and intact NaPi-IIa may not only occur via internalization and lysosomal degradation but might also involve release of vesicles containing the transporter and its fragments into urine. This possibility will require further investigation beyond the scope of this study.

Two major limitations of our study are that our preparation of bulk UEV did not further distinguish between types of UEVs and that we did not test human urine. The latter would require detailed metabolic analyses of healthy probands which was beyond the scope and possibilities of this study. Various recommendations have been made how to isolate, store and characterize UEVs [12, 47]. Our preparation represents a simplified isolation of bulk UEVs without further separation of small and large UEVs. Of note, similar preparations have been used from various species including humans to study the regulation of transport proteins [27–30, 34, 43, 48].

Taken together, two normalization methods, i.e. creatinine and TSG101 suggest that UEV replicate changes on the renal expression of the NaPi-IIa proteolytic fragment and of NaPi-IIc triggered by chronic changes in dietary Pi, whereas they provide contradictory results regarding the effects of acute dietary switches. This failure of UEV to replicate the acute adaptation of the renal cotransporters may at least partially explain the recently described lack of correlation of NaPi-IIa abundance between UEV and human nephrectomy samples [34]: normal diurnal fluctuations in Pi consumption prior to urinary collection may result in up or downregulation of the renal expression of NaPi-IIa that may not correlate with its UEV abundance. Moreover, it is unclear why the content of the NaPi-IIa proteolytic product in UEV reflects more faithfully than the full-length protein the downregulation of the cotransporter in renal BBM observed upon chronic Pi load. Although the abundance of both fragments was clearly reduced in BBM from rats chronically fed with high Pi compared with animals chronically fed with low Pi, the change was slightly more pronounced for the proteolytic fragment. This, together with potential degradation of the full-length protein during the 12 h of urine collection, could have masked changes at the level of the intact transporter.

## Supplementary Information

Below is the link to the electronic supplementary material.

Supplementary file1 (PDF 546 KB)

## Data Availability

All data are included, see also supplementary data.
