# Supplementary material for: Does the composition of urinary extracellular vesicles reflect the abundance of renal Na+/phosphate transporters?
Source: Pflugers Arch. 2022 Sep 8;474(11):1201–12. doi: 10.1007/s00424-022-02744-1 (PMC9560988; doi:10.1007/s00424-022-02744-1)

Supplementary Figure 1: LiCor protein staining of PVDF membranes incubated with NaPi-IIa and NaPi-IIc antibodies

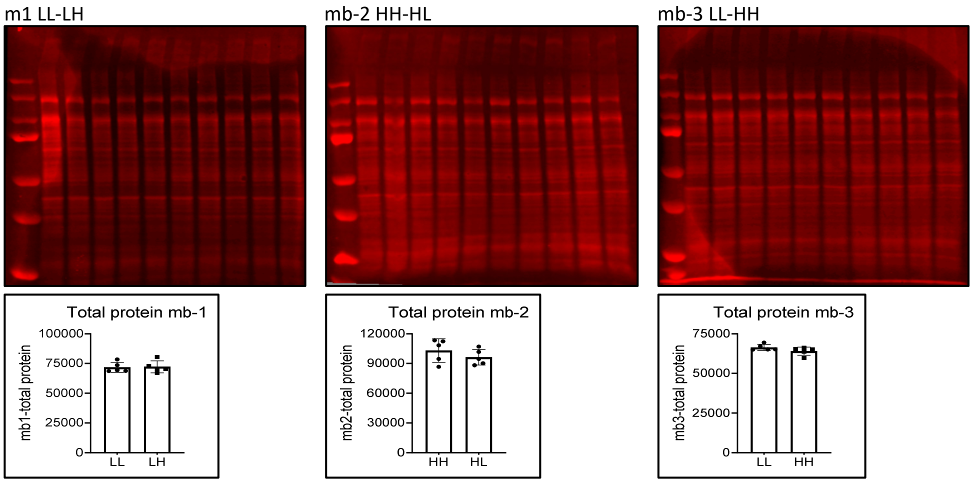

Supplementary Figure 2: LiCor protein staining of PVDF membranes incubated with AQP2 antibody

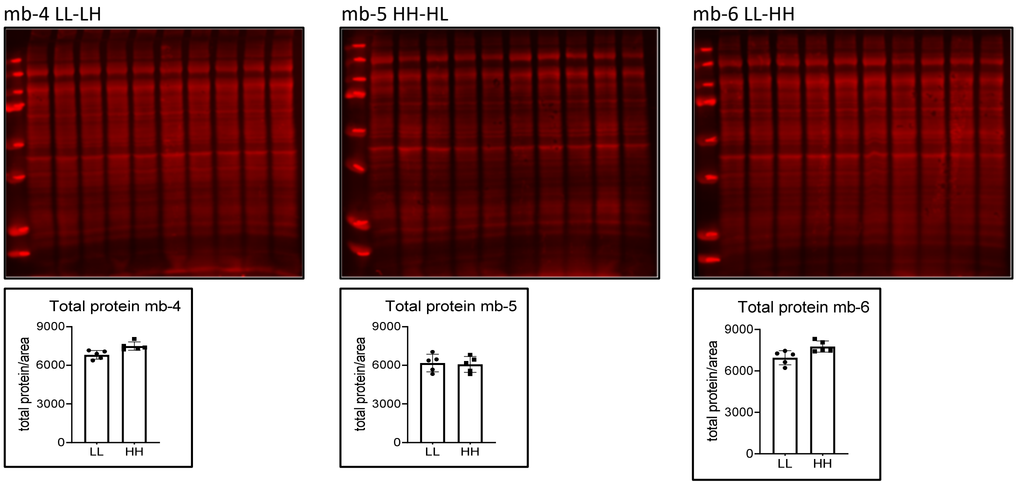

Supplement: Supplementary file 1 — Supplementary file1 (PDF 546 KB) [file 424_2022_2744_MOESM1_ESM.pdf]
